# Betalains in Edible Fruits of Three Cactaceae Taxa—*Epiphyllum*, *Hylocereus*, and *Opuntia*—Their LC-MS/MS and FTIR Identification and Biological Activities Evaluation †

**DOI:** 10.3390/plants10122669

**Published:** 2021-12-04

**Authors:** Michaela Barkociová, Jaroslav Tóth, Katarzyna Sutor, Natalia Drobnicka, Slawomir Wybraniec, Boris Dudík, Andrea Bilková, Szilvia Czigle

**Affiliations:** 1 Department of Pharmacognosy and Botany, Faculty of Pharmacy, Comenius University in Bratislava, Odbojárov 10, SK-832 32 Bratislava, Slovakia; barkociova2@uniba.sk; 2 Department of Chemical Engineering and Technology, Institute C-1, Faculty of Analytical Chemistry, Cracow University of Technology, ul. Warszawska 24, PL-31-155 Cracow, Poland; katarzyna.sutor@doktorant.pk.edu.pl (K.S.); nszmyr@chemia.pk.edu.pl (N.D.); slawomir.wybraniec@pk.edu.pl (S.W.); 3 Department of Cell and Molecular Biology of Drugs, Faculty of Pharmacy, Comenius University in Bratislava, Odbojárov 10, SK-832 32 Bratislava, Slovakia; dudik8@uniba.sk (B.D.); Andrea.Bilkova@uniba.sk (A.B.)

**Keywords:** *Epiphyllum*, *Hylocereus*, *Opuntia*, betalains, antioxidant activity, antimicrobial activity

## Abstract

*Epiphyllum*, *Hylocereus,* and *Opuntia* plants belong to the Cactaceae family. They are mostly known as ornamental plants but also for their edible fruits, which can potentially be sources of betalains, such as betanin, a natural pigment used in the food industry, e.g., under the European label code E 162. The aim of this work was the identification of betalains (using LC-MS/MS), evaluation of total betalain content (spectrophotometrically), analysis of functional groups (using FT-IR), evaluation of antioxidant activity (using DPPH, ABTS, FRAP, DCFH-DA, and reducing power methods) and evaluation of antimicrobial activity (*S. aureus*, *E. coli*, and *C. albicans*) in fruits of *Epiphyllum*, *Hylocereus*, and *Opuntia* taxa. A total of 20 betalains were identified in the studied Cactaceae fruits. The *Epiphyllum* pink hybrid had the highest values of total betalains amongst all samples. The highest antioxidant activity was observed in the *Epiphyllum* pink hybrid, in *Opuntia zacuapanensis* and *O. humifusa* fruits. The antimicrobial activity assay showed that cacti fruits were not able to effectively inhibit the growth of *E. coli*, *S. aureus*, or *C. albicans*. Our results prove that these fruits are good sources of natural pigments—betalains. They do not contain toxic compounds in significant amounts and they exhibit antioxidant activity.

## 1. Introduction

Species and hybrids of the genus *Epiphyllum* Haw. are cultivated and used mostly as ornamental plants. They are famous for their big, colourful, fragrant flowers [1], which usually bloom at night. Their fruits are plum-shaped and of various colours, typically shades of green to yellow or red to purple. They are edible, although usually not commercially available [2]. The toxicology and pharmacology of *Epiphyllum* plants have been of little scientific interest so far. To the best of our knowledge, there are no reliable sources regarding their biological activities, although there is a report on the moisturizing effect of *Epiphyllum oxypetalum* extract on human epidermal cells, which indicates its potential use in the cosmetic industry [3]. Phytochemical aspects of *Epiphyllum* plants were studied to a greater extent and various betalains, steroids, flavonoids, and other phenolic compounds were identified [1,4,5,6,7,8,9].

Species of the genus *Hylocereus* (A. Berger) Britton and Rose, syn. *Selenicereus* (A. Berger) Britton and Rose, are also used as ornamental plants due to their fragrant night-blooming flowers, but they are mostly famous for their big edible tropical fruits called pitaya, pitahaya, or dragon fruit. Individual species of this genus can be distinguished from each other by the colour of fruit skin and pulp, e.g., *Hylocereus monacanthus* (syn. *Hylocereus polyrhizus*, *Hylocereus lemairei*) is known as red pitaya because its flesh is red and the skin is pink, while *Hylocereus undatus*, known as white pitaya, has white pulp and pink skin. In folk medicine, *Hylocereus* flowers and stems (also called cladodes) have been used to treat cough, tuberculosis, bronchitis, mumps, diabetes, and as diuretic and wound-healing agents. Modern research proves some of these traditional indications as it suggests antioxidant, antiproliferative, antimicrobial, antidiabetic and antihyperlipidemic, anti-inflammatory, hepatoprotective, and wound-healing activities of *Hylocereus* plants. *Hylocereus* seed oil is gaining interest as a cosmetic ingredient. Studies of the phytochemical content of *Hylocereus* plants revealed the presence of betalains, flavonoids, phenolic acids and phenylpropanoids, terpenes and steroids, polysaccharides, and fatty acids [8,9,10,11,12].

*Opuntia* Mill. is the biggest and the best-known genus of the Cactaceae family. Fruits of *Opuntia* species are called prickly pear, tuna, or nopal. They are egg-shaped and of various colours, ranging from yellow to dark purple. However, the fruit is not the only edible part of these plants, as stems of *Opuntia* plants, called nopalitos, are also consumed in various forms as a vegetable [13,14]. Traditionally, *Opuntia* plants have been used to treat fatigue, rheumatism, gastric ulcers, facilitate healing of wounds, burns and insect bites, and as a diuretic [15,16]. Scientific research regarding their antioxidant, anti-inflammatory, antimicrobial, antiproliferative, antidiabetic, antihyperlipidemic, antiulcerogenic, wound healing, and diuretic activity is emerging [14,15,16,17,18]. The main phytochemical constituents of *Opuntia* plants are betalains, flavonoids, phenolic acids and phenylpropanoids, terpenes and steroids, polysaccharides, and fatty acids [13,14,15,16,17,18,19,20,21]. During drought periods, *Opuntia* stems (cladodes) can be used as a forage for animals and their mucilaginous tissue is also used in cosmetics, as is also their seed oil [9,10,14,17,19,20,21].

The Cactaceae family is known for its content of betalains, a class of plant-derived water-soluble natural pigments. Betalains can be subdivided into red-violet betacyanins and yellow-orange betaxanthins depending on their chemical structure. Betalains are not only safe to use as natural colourants, but their antioxidant, anticancer, antimicrobial, and antilipidemic activities suggest their vital role in human health and nutrition [22,23]. To this day, only betanin is used in the food industry under the name E 162 (beetroot red), but consumers’ demand for safe alternatives to synthetic dyes is growing. *Epiphyllum*, *Hylocereus*, and *Opuntia* fruits can be used as potential sources of these natural pigments, therefore it is important to know their betalain profile and their betalain content and to know to what extent pigments affect the biological activities of these fruits [24,25]. Screening of biological activities of *Epiphyllum*, *Hylocereus*, and *Opuntia* fruits is further important not only to understand their significance in human nutrition but also to find out whether these species could be used as medicinal plants.

The main aim of this study was the FTIR analysis, identification, and quantification of betalains in *Epiphyllum*, *Hylocereus*, and *Opuntia* fruits, the determination of their antioxidant and antibacterial activities, and its correlation with total betalain content.

## 2. Results and Discussion

### 2.1. Identification of Betalains

Betalains in Cactaceae fruits were identified using LC-MS/MS. The results of these analyses are summarized in Table 1 and Table 2. Due to the lack of plant material, only 19 samples were subjected to pigment identification.

The analysis revealed the presence of both types of betalains: red-violet betacyanins and yellow-orange betaxanthins. The most dominant compounds, present in all tested samples, were betanin, isobetanin, and phyllocactin.

Previous reports showed the presence of indicaxanthin, betanin, isobetanin, 4’-*O*-malonyl-betanin, phyllocactin, and isophyllocactin in *Epiphyllum* fruits. γ-Aminobutyryl acid-betaxanthin, 2’ apiosyl-betanin, 2’-apiosyl-isobetanin, 2’-*O*-apiosyl-phyllocactin, and 2’-*O*-apiosyl-isophyllocactin were previously reported only in flowers or stems, but not in fruits. However, the presence of hylocerenin and 4’-*O*-malonyl-isobetanin has not been reported earlier in *Epiphyllum* species [1,4].

All of the identified pigments have been reported previously in *Hylocereus* fruits [10]. Isobetanidin 5-*O*-β-sophoroside, 2’-apiosyl-betanin, 2’-apiosyl-isobetanin, 4’-*O*-malonyl-betanin, isogomphrenin I, isophyllocactin, 4’-*O*-malonyl-isobetanin, 6’-*O*-sinapoyl-glucosyl-betanin, and 6’-*O*-sinapoyl-glucosyl-isobetanin are probably reported in fruits of *Opuntia* species for the first time. The presence of other identified pigments has been revealed before [26,27].

### 2.2. Quantification of Betalains

Total betalain content of *Epiphyllum*, *Hylocereus* and *Opuntia* fresh fruit samples was evaluated spectrophotometrically and expressed as betanin (mg/g) in 26 samples (I–XXVI). Results are summarized in Table 3.

Total betalain content ranged from 0.00 to 5.09 mg/g. In two samples, *Hylocereus megalanthus* (V) and the green *Epiphyllum* hybrid (III), no absorption maxima were observed and therefore it was not possible to determine any betalain content. Out of all samples, pink (II) and violet (I) *Epiphyllum* hybrids had the highest values of total betalains (5.09 mg/g and 2.66 mg/g, respectively), followed by *Opuntia zacuapanensis* (XIX) and *Opuntia humifusa* (XVI) (2.34 mg/g and 2.33 mg/g, respectively). Higher betalain content was observed with dark fruits—violet, purple and red—while pink, rose, and orange fruits were characterized by lower betalain content.

According to sources of previous research, total betalain content in Cactaceae species varies greatly, which could be caused not only by the locality where the fruits were grown and harvested but also by methods used for betalain content quantification. For example, Erdelská and Stintzing reported total betalain content of 454.9 mg/kg and 255.7 mg/kg for the peel and flesh of a violet *Epiphyllum* hybrid, respectively [1]. Tang and Norziah determined the total betalain content in *Hylocereus polyrhizus* fleshy pulp water extract to be 9.8 mg/100 g, while the total betalain content of the peel of *Hylocereus undatus* reported by De Mello et al. was 101.04 mg/100 g [28,29]. When the total betalain content of 10 Mexican prickly pear cultivars was evaluated, it ranged from 0.17 to 8.15 mg/g, while the lowest value was reported for *Opuntia albicarpa* and the highest for *Opuntia robusta* [26].

### 2.3. FTIR Analysis

FTIR spectral analysis was used to determine the functional group in the dry methanolic extracts of *Epiphyllum*, *Hylocereus*, and *Opuntia* fruits. Table 4 shows the FTIR analysis results of plant samples of *Epiphyllum* (I–III), *Hylocereus* (IV–VII), and *Opuntia* (VIII–XX) taxa, as well as betanin (standard red beet extract diluted with dextrin, Sigma-Aldrich, USA). The peak of 3320–3335 cm^−1^ corresponds to the aromatic (R-NH-R containing) group stretch. The peaks of 1255–1259 cm^−1^ and 850–897 cm^−1^ represent C-O stretching vibrations, usually in a carboxylic acid. The peak at 1716–1725 cm^−1^ corresponds to the C=O stretching vibrations which represent the carbonyl group for ketone structures. The peak at 1590–1634 cm^−1^ represents C=N, at 1615 cm^−1^ it also contains the C=O group in RCO-OH (colourless samples III, V). The peak at 1031–1078 cm^−1^ represents C-N. The bands at 1403–1417 cm^−1^ and 920–929 cm^−1^ were attributed to the stretching vibration of the –OH bond. Our results are in accordance with literature data [30,31,32,33].

### 2.4. Antioxidant Activity

The antioxidant activity of *Epiphyllum*, *Hylocereus*, and *Opuntia* fresh fruit water extracts was evaluated using five different in vitro antioxidant assays. A total of 27 samples were tested. For the DPPH, ABTS, and NO scavenger assays, results were expressed as SC_50_-concentration of the sample extract providing 50% inhibition of a free radical. The lower the SC_50_ value, the higher the antioxidant activity. Results were compared with ascorbic acid, Trolox, and betanin solutions. For the FRAP and reducing power assays, results were expressed as the analogical amount of ascorbic acid (AA) at the initial sample concentration of 150 mg/mL, as well as compared with betanin (at the initial sample concentration of 20 mg/mL). The higher the AA value, the higher the antioxidant activity. SC_50_ and AA values are summarized in Table 5. Correlation coefficients between total betalain content and antioxidant activity are displayed in Table 6.

In the DPPH assay, the highest antioxidant activity was observed for *Opuntia humifusa* (XVI) (SC_50_ = 17.79 mg/mL), while the lowest was observed for two *Hylocereus* taxa (VI, VII) (SC_50_ = 798.15 mg/mL). It was not possible to determine the antioxidant activity of the betanin standard, as its mixture with DPPH showed flocculation. However, both the ascorbic acid water solution and the Trolox methanolic solution expressed SC_50_ values of 0.02 mg/mL.

In the ABTS assay, *Opuntia zacuapanensis* (XIX) showed the highest antioxidant activity (SC_50_ = 15.25 mg/mL), similar to the betanin water solution (SC_50_ = 16.78 mg/mL) while *Hylocereus megalanthus* (V) showed the lowest one (SC_50_ = 136.88 mg/mL). SC_50_ values for ascorbic acid water solution and Trolox ethanolic solution were 0.02 mg/mL and 0.28 mg/mL, respectively.

In the FRAP assay, the highest antioxidant activity (at the initial sample concentration of 150 mg/mL) was observed for the dark pink *Epiphyllum* hybrid (II) (AA = 7.79 μg/mL), while the lowest one was observed for *Hylocereus megalanthus* (V) (AA = 0.31 μg/mL). The AA value of betanin (at the initial sample concentration of 20 mg/mL) was 4.87 μg/mL.

According to the NO scavenger assay, *Opuntia tomentella* (XVIII) is the sample with the highest antioxidant activity (SC_50_ = 0.22 mg/mL), while *Hylocereus costaricensis* is the one with the lowest activity (SC_50_ = 21.35 mg/mL). SC_50_ values of betanin and ascorbic acid water solutions were 140.5 mg/mL and 9 × 10^−5^ mg/mL, respectively. Due to the flocculation of the mixture of the Griess reagent with both the ethanolic and methanolic Trolox solutions, it was not possible to determine Trolox antioxidant activity.

In the reducing power assay, the dark pink *Epiphyllum* hybrid (II) showed the highest antioxidant activity (AA = 3.27 μg/mL) at the initial sample concentration of 150 mg/mL, while the *Hylocereus* taxon with pink skin (VII) showed the lowest activity (AA = 0.32 μg/mL). The AA value of betanin (at the initial sample concentration of 20 mg/mL) was 4.15 μg/mL.

To the best of our knowledge, there are no reliable studies regarding the antioxidant activity of *Epiphyllum* hybrid fruits, therefore our study is the first of its kind. Table 6 shows the correlation coefficients between the total betalain content and the antioxidant activity of the selected *Epiphyllum*, *Hylocereus*, and *Opuntia* fruits.

The antioxidant activity of *Hylocereus* fruits was evaluated by various authors and methods. A DPPH assay of *Hylocereus* sp. flesh water extract conducted by Khalili et al. determined the SC_50_ value to be 1.45 mg/mL [34], and a DPPH assay of *Hylocereus undatus* ethanolic fruit extract determined the SC_50_ value to be 27.5 mg/mL [35]. When the ethanolic fruit extract of *Hylocereus undatus* was evaluated by an ABTS assay, its antioxidant activity was 1.57 ± 0.01 µmol Trolox/g, while it was 1.59 ± 0.04 µmol Trolox/g using the FRAP assay [36]. Another ABTS assay of *Hylocereus polyrhizus* fruit determined its antioxidant activity to be 30.5 ± 0.1 AAE mg/100 mL (ascorbic acid equivalents), while another FRAP assay of *Hylocereus* sp. showed the antioxidant activity of its ethanolic extract to be 1.24 ± 0.06 µmol Fe(II)/g [37,38]. To the best of our knowledge, no studies were conducted using the NO scavenger assay in *Hylocereus* sp. fruits. The reducing power assay was done on *Hylocereus polyrhizus* fruit juice and it showed that the reducing capability increased from 0.18 ± 0.02 in 0.03 g extract to 2.37 ± 0.18 in 0.50 g extract [39].

The antioxidant activity of *Opuntia* sp. is widely recognised and well documented in scientific literature. Several studies applied various methods in the assessment of the free radicals scavenging activity of *Opuntia* sp. fruits. A water extract of *Opuntia ficus-indica* fruits harvested during the summer season evaluated by the DPPH method showed SC_50_ = 45.10 ± 0.99 μg/mL. When evaluated by the FRAP method, results were expressed as μg FeSO_4_ eq/g of water extracts and the FRAP value was 1979.43 ± 29.33. The NO scavenging activity SC_50_ value of the same sample was 82.04 ± 0.09 μg/mL [40]. In another study, the DPPH and reducing power assays were employed to investigate the methanolic extract of *Opuntia ficus-indica* fruits; the DPPH assay SC_50_ value was 232.85 ± 1.87 µg/100 µL, while the reducing power assay resulted in an SC_50_ = 269.71 ± 1.09 µg/100 µL value [41]. Issaad et al. used a lyophilized fruit powder of *Opuntia ficus-indica* and the DPPH assay to determine its antioxidant activity; they report SC_50_ = 0.35 ± 0.01 mg/mL [42]. In *Opuntia elatior* fruits, the antioxidant activity of various extracts was measured using DPPH and NO scavenger activity; the ethylacetate fraction gave the best results, SC_50_ values of 44.52 ± 0.531 μg/mL and 51.08 ± 0.197 μg/mL, respectively [43]. A methanolic extract of *Opuntia joconostle* fruit showed antioxidant activity of 4.94 ± 0.64 mmol TE/100 g and 32.79 ± 1.42 mmol TE/100 g when the DPPH and ABTS assays were used, respectively; the reducing power assay of the same sample showed SC_50_ = 8.04 ± 0.52 mg/mL [44]. In another study, methanolic extracts of *Opuntia ficus-indica* were tested for antioxidant activity using DPPH, ABTS, and FRAP assays; the results, expressed as SC_50_ values (mg/mL) were 3.52 ± 0.03, 0.80 ± 0.05, and 8.04 ± 0.02, respectively [45]. When the antioxidant activity of *Opuntia dillenii* fruit extracts was evaluated using DPPH and ABTS assays, SC_50_ values ranged from 45 ± 3 μg/mL to 2000 μg/mL and from 88 ± 2 μg/mL to 2000 to μg/mL, respectively, depending on the extract used [46].

Because of the different approaches to antioxidant activity assays results expression, and because of different extraction methods used, it is complicated to compare the aforementioned results with our analyses. However, when DPPH, ABTS, and NO scavenger assays were used, and results expressed as SC_50_ values, we can see that the antioxidant activity of our samples was lower than of those reported previously. This could be explained by the fact that our samples were not cultivated and harvested in their natural tropical environment or by the fact that our extracts were made from fresh fruits directly and therefore were much less concentrated.

### 2.5. Antimicrobial Activity

Indicating bacteria and yeast differ in the cell structure, e.g., in the composition of their cell surfaces, which affect their permeability and can explain their different sensitivity to metabolites/bioactive constituents present in plant extracts. Both bacteria, *S. aureus* and *E. coli*, were highly susceptible to the commercial antibiotic ciprofloxacin (MIC 6.8 × 10^−4^ mM and 3 × 10^−4^ mM for *S. aureus* and *E. coli*, respectively), which was used as a positive antimicrobial agent with broad-spectrum activity for both Gram-positive and Gram-negative bacteria. As the tested samples represented fresh cactus juices pressed from *Epiphyllum*, *Hylocereus*, and *Opuntia* fruits with undefined chemical composition, we have evaluated their antimicrobial activity in percentage (*v*/*v*), not in units of concentration (M or g/L), as is typical. Our obtained results indicate that none of the tested extracts inhibited the growth of *S. aureus*, *E. coli*, or the yeast *C. albicans* even in the highest concentration tested (50% extract). The use of aqueous crude extracts without any prior purifying (except for gauze filtration) or concentrating procedures could explain the ineffectiveness of cacti extracts against all microorganisms tested.

## 3. Materials and Methods

### 3.1. Plant Material

Fruits of *Epiphyllum* Haw. hybrids—violet, pink and green—came from a private garden in Modra, Slovakia. Fruits of *Hylocereus* (Berger) Britt. species—*Hylocereus costaricensis* (red flesh, pink skin), *Hylocereus megalanthus* (white flesh, yellow skin), *Hylocereus undatus* (white flesh, pink skin), and *Hylocereus* sp. (red flesh, pink skin, species not identified by dendrologist)—were collected in the botanical garden “Fűvészkert” in Szeged, Hungary. Fruits of *Opuntia* Mill. species were obtained from the Comenius University Botanical Garden in Bratislava, Slovakia (*Opuntia aurea*, *Opuntia camanchica*, *Opuntia fragilis*, *Opuntia humifusa*, *Opuntia polyacantha*, and seven samples of *Opuntia* sp. not identified by dendrologist) and from the University Botanical Garden in Pécs, Hungary (*Opuntia crinifera*, *Opuntia tomentella*, and *Opuntia zacuapanensis*). Herbarium samples were frozen and deposited at the Department of Pharmacognosy and Botany (Comenius University in Bratislava, Slovakia) prior to analysis. Samples are listed in Table 7 with a summarization of their colour description, year of collection, and abbreviation used.

### 3.2. Preparation of Plant Extracts

For antioxidant activity determination, 15 g of fresh fruit samples were cut into small pieces and extracted three times with a total of 100 mL of distilled water using sonication for 10 min. For the spectrophotometric betalain quantification, these 15% (m/m) extracts were further diluted with distilled water to 10% (m/m).

For betalains identification and for the FTIR analysis, 10 g of fresh fruit samples were cut into small pieces and extracted three times with a total of 100 mL of a methanol/water mixture (60/40) using sonication for 10 min. The extracts were then evaporated to dryness under vacuum.

### 3.3. Identification of Betalains by LC-MS/MS

Prior to the analysis, dry extracts of samples were dissolved in 2 mL of distilled water and centrifuged at 3000× *g* rpm for 5 min, using a micro-centrifuge type 320 UNIPAN; 200 μL of each supernatant was used for analysis. For the identification of betalains, an LC-MS-8030 mass spectrometric system (Shimadzu, Kyoto, Japan) coupled to LC-20ADXR HPLC pumps, an injector model SIL-20ACXR, and a PDA detector model SPD-M20A was used, controlled with LabSolutions software version 5.60 SP1 (Shimadzu, Japan). Samples were eluted through a 150 mm × 4.6 mm i.d., 5.0 μm, Kinetex C18 chromatographic column preceded by a guard column of the same material (Phenomenex, Torrance, CA, USA). The injection volume was 40 μL and the flow rate was 0.5 mL/min. The column was thermostatted at 40 °C. Separation of the analytes was performed with a binary gradient system. The mobile phases were: A—2% formic acid in water, and B—pure methanol. The gradient profile was: (t (min); % B), (0; 5), (12; 30), (15; 80), (15.51; 5), and (19; 5). The full range of PDA signal was recorded, and chromatograms at 538 nm were individually displayed. Positive ion electrospray mass spectra were recorded by the LC-MS system which was controlled with LabSolutions software. The ionisation electrospray source operated in positive mode (ESI^+^) at an electrospray voltage of 4.5 kV, the capillary temperature at 200 °C and using N_2_ as a sheath gas, recording total ion chromatograms, mass spectra, and ion chromatograms in selected ion monitoring mode (SIM).

### 3.4. Quantification of Betalains

Quantification of betalains was performed in triplicate using a spectrophotometric method previously described by Castellanos-Santiago and Elhadi [26]. The UV/VIS absorption spectra of the samples’ 10% water extracts were recorded from 290 to 700 nm to obtain absorption values at their respective absorption maxima using a GENESYS™10 spectrophotometer (Thermo Electron Corporation, Cambridge, UK). The betalain content was expressed as betanin and was calculated using the samples’ absorption values at 538 nm and betanin molar weight (550.5 g/mol) and molar attenuation coefficient ε (60,000 L × mol^−1^ × cm^−1^).

### 3.5. FTIR

The infrared spectroscopy analyses of *Epiphyllum*, *Hylocereus*, and *Opuntia* fruits’ dry methanolic extracts were performed on a Nicolet FT-IR 6700 spectrometer (Thermo Electron Corporation, Cambridge, UK) with Omnic™ Spectra Software (Thermo Scientific, Waltham, MA, USA), in attenuated total reflectance sampling (ATR) mode. The infrared spectrum was measured in the mid-infrared region (4000–600 cm^−1^). The position and intensity of the absorption bands in the FTIR spectra were used to analyse the functional groups according to libraries and bibliography [30,31,32,33].

### 3.6. Antioxidant Activity

Antioxidant activity of *Epiphyllum*, *Hylocereus*, and *Opuntia* fruit extracts was determined using five different spectrophotometric methods. All measurements were performed on a GENESYS™10 spectrophotometer (Thermo Electron Corporation, Cambridge, UK). All chemicals used were of analytical grade.

#### 3.6.1. DPPH Method

The DPPH assay was conducted according to the method reported by Bilusic Vundac et al. [47]. An amount of 1.8 mL of DPPH methanol solution was added to 0.2 mL of various concentrations of fruit extracts. The solution was then thoroughly shaken and left to react in the dark at room temperature. The absorbance of the solution was measured after 30 min. Methanol (1.8 mL) plus plant extracts (0.2 mL) were used as blank; DPPH solution (1.8 mL) plus methanol (0.2 mL) was used as negative control; and positive control was 1.8 mL of DPPH solution plus 0.2 mL of ascorbic acid/Trolox solution. Antioxidant activity (%) was calculated using the samples’ vs. negative control’s absorption values at 517 nm, and results were expressed as SC_50_ (concentration of sample extract providing 50% inhibition of the DPPH radical). The assay was carried out in triplicate.

#### 3.6.2. ABTS Method

The ABTS assay was conducted according to the method reported by Re et al. [48]. An amount of 2 mL of ABTS radical solution was added to 0.1 mL of various concentrations of fruit extracts. The solution was then thoroughly shaken and left to react in the dark at room temperature. The absorbance of the solution was measured after 5 min. Ethanol (2 mL) plus plant extracts (0.1 mL) were used as blank; ABTS solution (2 mL) plus methanol (0.1 mL) was used as negative control; positive control was 2 mL of ABTS solution plus 0.1 mL of ascorbic acid/Trolox/betanin solution. Antioxidant activity (%) was calculated using the samples’ vs. negative control’s absorption values at 734 nm, and results were expressed as SC_50_ (concentration of sample extract providing 50% inhibition of the ABTS radical). The assay was carried out in triplicate.

#### 3.6.3. FRAP Method

The FRAP assay was conducted according to the method reported by Benzie and Strain [49]. An amount of 3 mL of the FRAP reagent was added to 0.1 mL of various concentrations of fruit extracts. The solution was then thoroughly shaken and left to react in the dark at room temperature. The absorbance of the solution was measured after 5 min. The FRAP reagent was used as blank. Betanin was used as the positive control. Results were expressed as analogical amount of ascorbic acid (μg/mL) and calculated using the samples’ absorption values at 593 nm. The assay was carried out in triplicate.

#### 3.6.4. NO Scavenger Method

The nitric oxide scavenger assay was conducted according to the method reported by Marcocci et al. [50]. An amount of 1 mL of sodium nitroprusside solution was added to 4 mL of various concentrations of fruit extracts. The solution was then thoroughly shaken and incubated at 37 °C for 150 min. A total of 1.5 mL of this solution was then added to 0.9 mL of Griess reagent, shaken properly and left to react in the dark at room temperature. The absorbance of the solution was measured after 5 min. Distilled water (0.9 mL) plus plant extracts after incubation with sodium nitroprusside solution (1.5 mL) were used as blank; Griess reagent (0.9 mL) plus distilled water after incubation with sodium nitroprusside (1.5 mL) was used as negative control; and positive control was 0.9 mL of Griess reagent plus 1.5 mL of ascorbic acid/betanin solution after incubation with sodium nitroprusside. Antioxidant activity (%) was calculated using the samples’ vs. negative control’s absorption values at 570 nm, and results were expressed as SC_50_ (concentration of sample extract providing 50% inhibition of the NO radical). The assay was carried out in triplicate.

#### 3.6.5. Reducing Power Method

The reducing power assay was conducted according to the method reported by Oktay et al. [51]. An amount of 0.5 mL of various concentrations of fruit extracts were mixed with 1.25 mL of phosphate buffer (pH 6.6) and 1.25 mL of potassium ferricyanide solution (1%). The solution was then thoroughly shaken and incubated at 50 °C for 20 min. Then, 1.25 mL of trichloroacetic acid (10%) was added to the mixture and shaken properly. Afterwards, 1.25 mL of this mixture was mixed with distilled water (1.25 mL) and 0.25 mL of ferric chloride solution (0.1%). The solution was then thoroughly shaken and left to react in the dark at room temperature. The absorbance of the solution was measured after 5 min. The reagent mixture (without fruit extracts) was used as blank; betanin was used as the positive control. Results were expressed as analogical amount of ascorbic acid (μg/mL) and calculated using the samples’ absorption values at 700 nm. The assay was carried out in triplicate.

### 3.7. Antimicrobial Activity

The antimicrobial activity of plant extracts was evaluated in vitro using the standard broth dilution method [52] with some modifications. The following strains of Gram-positive, Gram-negative bacteria and a yeast pathogen were selected for the experiments: *S. aureus* CNCTC Mau 82/78, *E. coli* CNCTC 327/73, and *C. albicans* CNCTC 59/91, respectively. All microbial strains were purchased from the Czech National Collection of Type Cultures, Czech Republic, and are recommended for antimicrobial susceptibility [53] and preservative efficacy tests [54]. Pressed cactus juices were prepared immediately before use. Working test solutions were prepared by serial dilution of cactus juices in sterile double-concentrated Nutrient broth (Imuna, Slovakia) or Sabouraud broth (Difco, France) for bacteria or yeast, respectively. The final concentration of tested extracts in the samples ranged from 50% to 0.1% (*v*/*v*). Each concentration was assayed in triplicates. For each plant extract and microorganism, the following controls were used: blank, uninoculated media without test extract to account for changes in the media during the experiment; negative control, uninoculated media containing only the test extract; positive control, inoculated media without extract. The minimal inhibitory concentration (MIC) was defined as the highest dilution (the lowest percentage/concentration) of plant extract inhibiting the growth of microorganisms on agar plates for all parallel samples compared with the positive control after 24 h.

### 3.8. Statistical Analysis of Data

All the measurements were done in three replications. Results were mean values of multiple repetitions standard deviation (SD). The results were compared with the control and reference groups. Statistical analysis with ANOVA (Statistica 13.0, StatSoft Inc., Tulsa, OK, USA) was used. The statistical relationship between the antioxidant activities and betalain content was evaluated according to Pearson’s correlation test.

## 4. Conclusions

Cactaceae fruits are mostly recognized and consumed as edible tropical fruits, but they are also used in folk medicine. They contain betalains, a class of plant-derived nitrogen-containing natural pigments, and also flavonoids, phenolic acids and phenylpropanoids, fatty acids and terpenes. Some Cactaceae family taxa—*Epiphyllum*, *Hylocereus*, and *Opuntia*—were studied in this work. Our results prove *Epiphyllum*, *Hylocereus*, and *Opuntia* fruits are a good source of natural pigments—betalains. A total of 20 compounds were identified in the 26 chosen plant samples. The fruits do not contain any toxic compounds in significant amounts, and they exhibit antioxidant activity. The antimicrobial activity assay showed that none of the cacti fruits were able to inhibit the growth of *E. coli*, *S. aureus*, or *C. albicans*.

## Figures and Tables

**Table 1:** Chromatographic, spectrophotometric, and mass spectrometric data of betalains identified in dry extracts of *Epiphyllum*, *Hylocereus*, and *Opuntia* fruits.

No.	Compound	Rt [min]	λmax [nm]	*m*/*z* [M+H]+	*m*/*z* from MS/MS of [M+H]+
1	γ-aminobutyryl acid-betaxanthin	7.7	465	297	253
2	Indicaxanthin	8.4	478	309	265
3	Betanidin 5-*O-β*-sophoroside	9.0	n.d.	713	n.d.
4	Betanin	9.1	534	551	389
3′	Isobetanidin 5-*O-β*-sophoroside	9.8	n.d.	713	n.d.
4’	Isobetanin	9.9	534	551	389
5	2’-apiosyl-betanin	10.4	534	683	551; 389
5’	2’-apiosyl-isobetanin	11.1	534	683	551; 389
6	Gomphrenin I	11.2	538	551	389
7	Phyllocactin	11.3	533	637	593; 551; 389
8	4’-*O*-malonyl-betanin	11.6	533	637	593; 551; 389
6’	Isogomphrenin I	11.8	538	551	389
7’	Isophyllocactin	11.9	533	637	593; 551; 389
9	Hylocerenin	12.3	534	695	651; 551; 389
8’	4’-*O*-malonyl-isobetanin	12.4	533	637	593; 551; 389
10	2’-*O*-apiosyl-phyllocactin	12.6	533	769	683; 551; 389
9’	Isohylocerenin	12.9	534	695	651; 551; 389
10’	2’-*O*-apiosyl-isophyllocactin	13.1	533	769	683; 551; 389
11	6’-*O*-sinapoyl-glucosyl-betanin	13.9	538	919	757; 551; 389
11’	6’-*O*-sinapoyl-glucosyl-isobetanin	14.2	538	919	757; 551; 389

**Table 2:** Betalains identified in dry extracts of *Epiphyllum*, *Hylocereus,* and *Opuntia* fruits.

Compound ^a^	Rt [min]	*m/z* [M+H]^+^	Plant Sample ^b^																		
			I	II	IV	VI	VII	VIII	IX	X	XI	XII	XIII	XV	XVI	XVII	XVIII	XIX	XXI	XXII	XXVI
1	7.7	297	+ ^c^	+	+		+					+		+	+		+	+			
2	8.4	309	+++	+	+	+	++	+	++			+++		+	++	+	++	+++	+	+	+
3	9.0	713						+				+				+				+	+
4	9.1	551	+++	+++	+++	+++	+++	+++	+++	++	+++	+++	++	+++	+++	+++	+++	+++	+++	++	+++
3’	9.8	713						+				+				+				+	+
4’	9.9	551	+++	++	++	+	++	+	++	++	++	++	++	++	++	++	++	++	+++	+	++
5	10.4	683		+	+	+	+		+			+									
5’	11.1	683		+	+	+	+		+			+									
6	11.2	551						+			+		+	+	+	+	+	+	+		+
7	11.3	637	+++	++	++	++	++	+++	+++	++	+	+	++	+	+	+	+	+	+	+++	+
8	11.6	637	+	+	+	+	+		+			+									
6’	11.8	551										+		+	+	+		+	+		
7’	11.9	637	+++	++	+	+	+	++	++	+	+			+	+	+				++	+
9	12.3	695	+				+		+												
8’	12.4	637	+	+		+			+			+									
10	12.6	769	+		+		+		+												
9’	12.9	695					+														
10’	13.1	769	+		+		+		+												
11	13.9	919										+									
11’	14.2	919										+									

^a^ Compounds: see Table 1; ^b^ plant samples: I–III: *Epiphyllum*, IV–VII: *Hylocereus*, and VIII–XXVI: *Opuntia* (taxa see Table 7); absent samples—fruits with white or green pulp: III, V, XIV, XX, XXIII, XXIV, and XXV; ^c^ relative content values: +++ high content; ++ medium content; + traces.

**Table 3:** Total betalain content of *Epiphyllum*, *Hylocereus*, and *Opuntia* fresh fruits.

Plant Sample ^a^	Total Betalain Content (mg/g)	Plant Sample	Total Betalain Content (mg/g)
I	2.66 ± 0.19	XIV	0.09 ± 0.00
II	5.09 ± 0.44	XV	1.25 ± 0.01
III	0.00 ± 0.00	XVI	2.33 ± 0.02
IV	0.47 ± 0.30	XVII	1.90 ± 0.01
V	0.00 ± 0.00	XVIII	1.19 ± 0.01
VI	0.17 ± 0.02	XIX	2.34 ± 0.02
VII	0.37 ± 0.03	XX	1.22 ± 0.01
VIII	0.19 ± 0.02	XXI	1.84 ± 0.01
IX	0.32 ± 0.03	XXII	0.12 ± 0.01
X	0.18 ± 0.01	XXIII	0.12 ± 0.01
XI	0.22 ± 0.02	XXIV	0.47 ± 0.03
XII	1.40 ± 0.01	XXV	0.09 ± 0.00
XIII	0.26 ± 0.02	XXVI	0.26 ± 0.02

^a^ Plant samples: I–III: *Epiphyllum*, IV–VII: *Hylocereus*, and VIII–XXVI: *Opuntia* (taxa see Table 7).

**Table 4:** FTIR spectral analysis of dry methanolic *Epiphyllum*, *Hylocereus*, and *Opuntia* fruits extracts.

	Wavenumber [cm^−1^]												
Plant Sample	R-NH-R + O-H [band overlap]	C-H	C=O	C=N	-OH	C-H	C-O [in RCO-OH]	C-N [in C-N-C]	C-N [in C-N-C]	C-N	C-H	-OH	C-O [in RCO-OH]
I	3327	2933	1723	1600	1410	–	1257	1050	1034	–	0993	923	867
II	3329	2928	1721	1590	1403	–	1257	1053	1031	–	–	920	867
III ^a^	3330 **O-H**	2939 **O-H**	1725 **C=O**	1615 **C=O** **[in RCO-OH]**	–	1390 **C-C** **[in aromatic group]**	1312 **C-C** **[in aromatic group]**	–	1052 **C-O** **[in RCO-OH**]	–	–	–	–
IV	3331	2927	–	1601	1417	1360	1258	–	1043	–	–	–	895
V ^a^	3334 **O-H**	2929 **O-H**	1725 **C=O**	1615 **C=O** **[in RCO-OH]**	–	1385 **C-C** **[in aromatic group]**	1311 **C-C** **[in aromatic group]**	–	1049 **C-O** **[in RCO-OH]**	–	–	–	–
VI	3334	2926	1719	1602	1412	–	1255	1101	1031	–	–	–	891
VII	3327	2924	–	1594	1413	1360	–	1074	1031	–	–	–	895
VIII	3327	2931	1716	1601	1409	–	–	1231	1102	–	993	925	–
IX	3332	2929	–	1601	1413	–	1259	–	1050	–	–	–	866
X	3335	2925	–	1621	1417	–	1256	1031	1029	–	–	–	897
XI	3331	2931	–	1609	1413	–	1249	1054	1048	–	–	–	865
XII	3320	2930	–	1609	1417	–	1261	1105	1047	–	992	925	832
XIII	3327	2929	1716	1598	1416	–	1250	–	–	–	992	926	832
XIV	3327	2929	–	1615	1417	–	1259	–	1029	–	–	–	897
XV	3334	2930	1723	1604	1416	–	1251	–	1050	–	–	922	867
XVI	3325	2930	–	1602	1416	–	1262	1105	1048	–	992	925	832
XVII	3332	2931	–	1606	1416	–	1257	–	1050	–	993	922	866
XVIII	3334	2931	1714	1615	1413	–	1255	1103	1049	–	993	924	867
XIX	3331	2932	1719	1614	1416	–	1259	1102	1050	–	993	924	867
XX	3327	2930	1713	1609	1413	–	1258	1103	1049	–		924	867
betanin ^b^	3337	2931	–	1634	–	1362	–	1149	1078	1022 ^b^ **[band overlap with dextrin]**	–	0929	850

^a^ The presence of betalains (nitrogen-containing plant pigments) could not be confirmed in the sample, the respective bands of the spectra are most probably associated with oxygen-containing functional groups typical to flavonoids and/or phenolic acids; ^b^ the betanin standard spectra probably showed a C-N band overlap with dextrin.

**Table 5:** Antioxidant activity of *Epiphyllum*, *Hylocereus*, and *Opuntia* fresh fruit water extracts.

Plant Sample ^a^	DPPH (SC_50_ mg/mL)	ABTS (SC_50_ mg/mL)	FRAP (AA μg/mL) ^b^	NO Scavenger (SC_50_ mg/mL)	Reducing Power (AA μg/mL) ^b^
I	29.10 ± 2.10	43.09 ± 4.00	5.71 ± 0.48	1.61 ± 0.08	2.57 ± 0.22
II	22.47 ± 2.00	34.38 ± 2.00	7.79 ± 0.77	8.43 ± 0.78	3.27 ± 0.23
III	165.84 ± 11.00	97.92 ± 8.22	1.11 ± 0.11	0.44 ± 0.03	0.76 ± 0.66
IV	552.98 ± 42.78	73.89 ± 6.78	0.82 ± 0.06	21.35 ± 2.04	0.90 ± 0.08
V	179.50 ± 12.45	136.88 ± 11.22	0.31 ± 0.02	6.46 ± 0.56	0.50 ± 0.04
VI	798.15 ± 60.10	67.64 ± 5.44	1.18 ± 0.11	7.76 ± 0.68	0.73 ± 0.06
VII	798.15 ± 62.22	67.64 ± 5.88	1.18 ± 0.12	2.02 ± 0.21	0.32 ± 0.02
VIII	293.90 ± 22.30	40.18 ± 4.00	1.44 ± 0.13	0.79 ± 0.08	0.66 ± 0.05
IX	272.55 ± 22.10	42.39 ± 4.21	1.53 ± 0.16	3.33 ± 0.02	1.32 ± 0.10
X	470.92 ± 40.22	40.71 ± 4.00	2.04 ± 0.22	8.08 ± 0.80	0.49 ± 0.00
XI	70.26 ± 6.22	16.29 ± 1.21	3.27 ± 0.32	3.51 ± 0.30	0.35 ± 0.03
XII	67.26 ± 5.22	18.47 ± 1.21	2.31 ± 0.22	0.49 ± 0.00	1.16 ± 0.10
XIII	146.53 ± 10.67	47.94 ± 4.22	2.32 ± 0.22	2.08 ± 0.22	0.64 ± 0.07
XIV	776.81 ± 65.22	49.16 ± 4.81	1.16 ± 0.11	1.43 ± 0.10	0.87 ± 0.06
XV	65.32 ± 5.44	39.75 ± 3.64	2.03 ± 0.19	4.77 ± 0.42	1.26 ± 0.10
XVI	17.79 ± 1.28	32.21 ± 3.02	5.37 ± 0.51	9.84 ± 0.88	1.63 ± 0.12
XVII	32.04 ± 3.02	17.94 ± 1.42	5.24 ± 0.48	3.92 ± 0.34	1.16 ± 0.09
XVIII	53.34 ± 4.22	19.74 ± 2.00	3.46 ± 0.32	0.22 ± 0.02	0.61 ± 0.05
XIX	57.79 ± 4.67	15.28 ± 1.32	1.98 ± 0.14	1.71 ± 0.01	0.71 ± 0.05
XXVI	91.65 ± 8.66	20.21 ± 2.00	2.51 ± 0.21	7.12 ± 0.05	0.47 ± 0.03
XX	55.41 ± 4.33	40.03 ± 4.00	2.65 ± 0.18	8.20 ± 0.07	1.69 ± 0.12
XXI	44.06 ± 3.56	31.04 ± 3.00	4.48 ± 0.43	0.50 ± 0.04	1.18 ± 0.08
XXIV	211.22 ± 18.66	38.67 ± 3.20	1.85 ± 0.15	4.00 ± 0.30	0.66 ± 0.05
XXII	754.98 ± 67.45	50.44 ± 5.21	1.35 ± 0.11	0.87 ± 0.07	0.62 ± 0.04
XXIII	368.79 ± 32.56	60.91 ± 5.88	1.54 ± 0.08	5.30 ± 0.44	0.69 ± 0.04
XXV	230.74 ± 20.11	36.20 ± 3.24	1.61 ± 0.09	3.44 ± 0.22	0.52 ± 0.04
Betanin	–	16.78 ± 1.45	4.87 ± 0.32 ^c^	140.50 ± 10.22	4.15 ± 0.33 ^c^
Ascorbic acid	0.02 ± 0.00	0.02 ± 0.00	–	9 × 10^−5^	–
Trolox	0.02 ± 0.00	0.28 ± 0.00	–	–	–

^a^ Plant samples: I–III: *Epiphyllum*, IV–VII: *Hylocereus*, and VIII–XXVI: *Opuntia* (taxa see Table 7); ^b^ AA value at the initial sample concentration of 150 mg/mL; ^c^ AA value at the initial sample concentration of 20 mg/mL.

**Table 6:** Correlation coefficients between total betalain content and antioxidant activity of *Epiphyllum*, *Hylocereus*, and *Opuntia* fruits.

Plant Taxon	DPPH	ABTS	FRAP	NO Scavenger	Reducing Power
*Epiphyllum* hybrids	–0.90	–0.93	**0.98**	**0.91**	**0.97**
*Hylocereus* sp.	–0.81	–0.75	0.58	0.55	0.34
*Opuntia* sp.	–0.63	–0.55	0.70	0.08	0.60
All samples	–0.16	–0.40	**0.87**	–0.03	**0.86**

**Table 7:** List of *Epiphyllum*, *Hylocereus*, and *Opuntia* fruit samples.

Abbreviation	Plant Sample	Year of Collection	Colour Description
I	*Epiphyllum* hybrid	2012	Violet
II	*Epiphyllum* hybrid	2012	Dark pink
III	*Epiphyllum* hybrid	2012	Green
IV	*Hylocereus costaricensis*	2012	Red pulp, Pink skin
V	*Hylocereus megalanthus*	2012	White pulp, Yellow skin
VI	*Hylocereus undatus*	2012	White pulp, Pink skin
VII	*Hylocereus* sp.	2018	Red pulp, Pink skin
VIII	*Opuntia aurea*	2016	Light orange
IX	*Opuntia camanchica*	2016	Pink
X	*Opuntia camanchica*	2017	Rose
XI	*Opuntia camanchica*	2018	Light rose
XII	*Opuntia crinifera*	2018	Red
XIII	*Opuntia fragilis*	2016	Pink
XIV	*Opuntia humifusa*	2016	Light rose
XV	*Opuntia humifusa*	2017	Dark violet
XVI	*Opuntia humifusa*	2018	Dark violet
XVII	*Opuntia polyacantha*	2016	Dark violet
XVIII	*Opuntia tomentella*	2018	Dark red
XIX	*Opuntia zacuapanensis*	2018	Dark red
XX	*Opuntia* sp.	2012	Dark violet
XXI	*Opuntia* sp.	2018	Purple
XXII	*Opuntia* sp.	2018	Light orange
XXIII	*Opuntia* sp.	2012	Orange
XXIV	*Opuntia* sp.	2013	Dark red
XXV	*Opuntia* sp.	2015	Dark orange
XXVI	*Opuntia* sp.	2016	Pink

## Data Availability

The data presented in this study are available on request from the corresponding author.
